# Expression pattern divergence of duplicated genes in rice

**DOI:** 10.1186/1471-2105-10-S6-S8

**Published:** 2009-06-16

**Authors:** Zhe Li, He Zhang, Song Ge, Xiaocheng Gu, Ge Gao, Jingchu Luo

**Affiliations:** 1 College of Life Sciences, National Laboratory of Plant Genetic Engineering and Protein Engineering, Center for Bioinformatics, Peking University, Beijing 100871, PR China; 2 State Key Laboratory of Systematic and Evolutionary Botany, Institute of Botany, Chinese Academy of Sciences, Beijing 100093, PR China

## Abstract

**Background:**

Genome-wide duplication is ubiquitous during diversification of the angiosperms, and gene duplication is one of the most important mechanisms for evolutionary novelties. As an indicator of functional evolution, the divergence of expression patterns following duplication events has drawn great attention in recent years. Using large-scale whole-genome microarray data, we systematically analyzed expression divergence patterns of rice genes from block, tandem and dispersed duplications.

**Results:**

We found a significant difference in expression divergence patterns for the three types of duplicated gene pairs. Expression correlation is significantly higher for gene pairs from block and tandem duplications than those from dispersed duplications. Furthermore, a significant correlation was observed between the expression divergence and the synonymous substitution rate which is an approximate proxy of divergence time. Thus, both duplication types and divergence time influence the difference in expression divergence. Using a linear model, we investigated the influence of these two variables and found that the difference in expression divergence between block and dispersed duplicates is attributed largely to their different divergence time. In addition, the difference in expression divergence between tandem and the other two types of duplicates is attributed to both divergence time and duplication type.

**Conclusion:**

Consistent with previous studies on *Arabidopsis*, our results revealed a significant difference in expression divergence between the types of duplicated genes and a significant correlation between expression divergence and synonymous substitution rate. We found that the attribution of duplication mode to the expression divergence implies a different evolutionary course of duplicated genes.

## Background

Gene and genome duplications have long been recognized as the primary source for evolutionary novelties [1,2]. Observations on eukaryotic genomes suggest that gene duplications arise at a high rate which is relatively uniform across species [3]. Furthermore, recent studies have revealed that whole genome duplication (polyploidy) has been rampant in the evolutionary course of eukaryotes, particularly the angiosperm lineage of plants [4]. It is estimated that the incidence of polyploidy of angiosperms is 30–80%, and ploidy changes may represent for 2–4% of speciation events [5]. Analyses of whole genome sequences have shown that polyploidy has occurred repeatedly in plants even with small genome size, such as *Arabidopsis thaliana *[6-8]. Theoretical models suggest four possible fates for duplicated genes: (1) gene loss (nonfunctionalization) [1,3,5,9], (2) acquirement of novel beneficial functions (neofunctionalization) [1,2], (3) functional complementary (subfunctionalization) [10,11], (4) functional buffering (redundancy) [12-14].

It has also been proposed that expression divergence is crucial for the retention of duplicated genes in a genome, and considered as very influential on protein [15] and morphological evolution [16]. With the availability of expression data it is possible to draw a global picture of expression divergence of duplicated genes. In yeast, Gu *et al. *[17] found a significant positive correlation between expression divergence and sequence divergence measured by estimated rate of synonymous substitutions (*K*_S_) and nonsynonymous substitutions (*K*_A_). Based on the correlation between duplicated gene pairs with *K*_A _less than 0.3, they proposed that the expression pattern of most duplicated genes quickly diverged after duplication events. Using Affymetrix expression data, Makova and Li [18] analyzed the expression pattern of human duplicated genes and found a positive correlation between expression divergence and *K*_S _or *K*_A _when *K*_A _< 0.2, in agreement with Gu *et al. *[17].

Recently, studies on evolution of expression patterns of duplicated genes in the *Arabidopsis *genome have been reported. Blanc and Wolfe [19] showed that more than half of the recently duplicated genes in *Arabidopsis *diverged in expression patterns and a significant correlation existed between sequence similarity and expression divergence of those duplicates. However, using massively parallel signature sequencing (MPSS) dataset, Haberer *et al. *[20] found that no significant correlation existed between expression divergence and divergence time of duplicated genes. On the other hand, recent studies have demonstrated that the pattern of expression divergence is different between genes derived from whole genome and small-scale duplications. Casneuf *et al. *[21] reached the conclusion that duplicated genes identified as remnants of large-scale duplications had more correlated expression patterns than those created by small-scale duplications, and the former tended to have highly redundant or overlapping expression patterns. Using MPSS and microarray data of *Arabidopsis*, Ganko *et al. *[22] systematically analyzed the expression pattern divergence of gene pairs derived from segmental, tandem and dispersed duplications. They found the expression patterns of tandem and dispersed duplicated genes diverged quickly soon after duplication events, and there was a strong positive relationship between expression divergence and *K*_A_, but not between expression divergence and *K*_S_. They found that about 70% of gene pairs had asymmetric expression divergence where one gene was expressed at a higher level across all assayed conditions. Chapman *et al. *[14] systematically analyzed SNPs in the *Arabidopsis *and rice genomes, and found that SNPs in duplicated genes encoded less radical amino acid changes than those in singleton genes. In addition to the observations that genes encoding long and complex proteins were more likely to preserve, they proposed that buffering of crucial functions may be the primary advantage of retention of duplicated genes.

As one of the most important alimentary crops, rice has served as a model plant of monocotyledons which is distinct from the dicotyledonous model plant *Arabidopsis *in many aspects [23]. The complexity of the structure of the rice genome has been demonstrated by its whole genome sequence [24,25]. Recent studies have classified rice as an ancient polyploid by the identification of a large set of duplicated blocks which are recognized as the remnants of whole genome duplication events [26-29]. Availability of large-scale expression data of the rice genome makes it an attractive model plant for exploring the evolution of duplicated genes. To date, systematic investigation on the divergence of expression pattern of rice duplicated genes using genome-wide microarray data has not been reported. In this work, we classified all duplicated gene pairs into three types (block, tandem and dispersed pairs) according to the different duplication modes, as suggested by Ganko *et al*. [22]. Block pairs were duplicated gene pairs located in collinear blocks derived by large-scale duplications. Gene pairs derived by small-scale duplications were subdivided into tandem pairs with the two members closely located on the same chromosome, and dispersed pairs contained all other duplicates which were neither closely located nor lying in the collinear blocks. The three types of duplicated genes showed a significant difference in expression divergence. We further revealed a significant correlation between expression divergence and synonymous substitution rate *K*_S _which has been widely used as an indicator of divergence time. Thus both duplication type and divergence time had influence on the diverged expression pattern among duplicated genes. To assess the attribution of these two factors, we applied a linear model to the data. Our results suggested that the different expression correlation between block and dispersed duplicated genes was largely attributed to their different synonymous substitution rates. On the other hand, the different expression correlation between tandem and the other two types of duplicates was not only attributed to their different *K*_S_, but also their different duplication types.

## Materials and methods

### Identification of duplicated gene pairs in three classes

All rice (*Oryza sativa *ssp. *japonica*) genes were obtained from the TIGR rice genome annotation version 5 . We excluded all transposable element-related genes. To select duplicated gene pairs, we performed an all-against-all protein sequence similarity search using BLASTP [30] with default parameters. We applied the criteria proposed by Gu *et al. *[31] to assign duplicated pairs: (1) the alignable region between the two sequences should be longer than 80% of the longer one; (2) the identity between the two sequences (*I*) should be *I *≥ 30% if the alignable region is longer than 150 a.a. and *I *≥ 0.01*n *+ 4.8*L*^-0.32(1+exp(-*L*/1000)) ^if otherwise, where *L *is the alignable length between the two sequences [32]. Here, we set *n *= 6 which makes the formula continuous at *L *= 150. Then we detected collinear blocks in the rice genome using the method described in [33] with parameters *mg *= 76 and *r *= 4, and found 501 significant collinear blocks among which 21 blocks containing more than 14 collinear gene pairs were selected as 'long blocks'. According to the chromosomal position of duplicated genes, we further assigned duplicated gene pairs into three classes: (*i*) block pairs which reside in the long blocks, (*ii*) tandem pairs with both members lying in the same chromosome and the number of intervening genes less than *mg*, and (*iii*) dispersed pairs which included all other duplicated pairs except for those located in the significant blocks. In the case of potential false positive, we discarded duplicated gene pairs located in short significant collinear blocks because of the ambiguity of their evolutionary sources (large-scale or small-scale duplications).

### Sequence analysis of duplicated gene pairs

We aligned the protein sequences of duplicated gene pairs by CLUSTALW [34], and used these alignments to guide corresponding coding nucleotide sequence alignments. *K*_S _estimates were obtained using the CODEML program [35] of the PAML package (version 4b) [36]. Codon frequencies were estimated by the empirical frequency of codon usage across the alignment, based on the observation of preferential codon usage in the rice genome [37]. As suggested by Casneuf *et al. *[21], we repeated calculations five times with different initial *κ *values (0.5, 1.0, 1.5, 2.0, 3.0) and used the estimates with maximum likelihood as the final estimates of *K *values to avoid incorrect estimates caused by local optimum. We discarded gene pairs with estimated *K*_S _> 4 for further analysis to reduce the influence of potential substitution saturation [38].

### Expression data analysis

Our analysis was based on the microarray expression data generated by Affymetrix rice genome array with various tissues/organs including seedling root, mature leaf and young leaf, and different stages of reproductive development such as panicle and seed [39]. The raw data containing 45 slides for 15 samples with three replicates for each sample were downloaded from the NCBI Gene Expression Omnibus (GEO dataset ID GSE6893) [40]. First, we assessed the quality for all 45 arrays using the affyPLM package [41] of Bioconductor [42] and discarded one slide (GSM159192) because of the possible artefact, implied by the broken image. Expression measures for each probe set were obtained by the robust multi-array average (RMA) method [43] implemented in Bioconductor. Presence/Absence (P/A) calls for all probe sets in each slide were made by the MAS5 algorithm. We took a probe set to "be present in a sample" if and only if present calls were assigned for all replicates of that sample. Each probe set was assigned to the gene model according to Affymetrix annotation. We discarded genes with multiple splicing isoforms expressed in one or more samples. Then we calculated the absolute and relative numbers of samples in which each member of the duplicated gene pairs were expressed. The relative expression number was the ratio of the number of samples in which a gene was present, to the total number of samples the gene pair was expressed [21]. The absolute expression number indicates the expression breath of duplicated genes and the relative expression number is a proxy of expression identity [21]. To measure the expression divergence of two duplicated genes, the Spearman correlation coefficient *ρ *was calculated for each pair accordingly.

### Regression analysis

To evaluate the attribution of duplication mode and *K*_S _to the expression divergence between duplicated genes, we applied a simple linear model to fit the data:

(1)

Here, *Y *is the transformed expression correlation log [(1+*ρ*)/(1-*ρ*)] which has a more appropriate scale for linear regression in comparison with the original correlation coefficient *ρ *[17], *K*_S _is the estimate of synonymous substitution rate, *I*_B _and *I*_T _are dummy variables to indicate duplication class, *X*_B _and *X*_T _are interaction of *K*_S _and duplication class, *ε *is the random error. We set



First, we applied the ordinary least square estimation to fit the modal and evaluate the significance of each regression coefficient by *t*-test. To mitigate the influence of violation of restrict assumptions of linear model and potential outliners, we applied MM-estimation [44] to obtain robust estimates of regression coefficients. Then we calculated 95% percentile confidence intervals for these regression coefficients by non-parametric bootstrap (the number of replicates = 5,000). Taking randomness of the predictors into consideration, we performed case resampling but not residual resampling [45]. The significance of regression coefficients was assigned by the corresponding 95% confidence intervals. Furthermore, we performed local regression to explore the local trend between the expression correlation and the *K*_S _values using the R locfit package.

For gene families containing more than two genes, it is possible for a gene to be present in more than one gene pairs, which violates the independent distributed assumption of the linear model. Therefore, we applied Spearman correlation coefficient, robust regression and bootstrap to mitigate the unduly influence of such violation of model assumptions. We also selected independent pairs proposed by Gu *et al. *[17] to investigate the influence of correlation of duplicated pairs. For each gene family, we first selected the pair with the smallest *K*_S _> 0.01, then chose pairs with increasing *K*_S _which did not share genes in common with other selected pairs. We performed all the analyses to the independent pairs.

## Results

### Expression pattern divergence varies with duplication mode

A total number of 3,306 duplicated gene pairs were identified from the *Oryza sativa *ssp. *japonica *genome. Based on the statistical approach proposed previously [33], we detected and evaluated collinear blocks which corresponded to the large-scale duplication events. The whole set of duplicated genes were further divided into 716 block pairs, 849 tandem pairs and 1,741 dispersed pairs according to their duplication modes. Block pairs that were still lying in recognizable duplicated collinear segments were regarded as pairs derived by large-scale duplication events. Tandem pairs and dispersed pairs were referred to small-scale duplications and created by different mechanisms [46].

**Figure 1:**
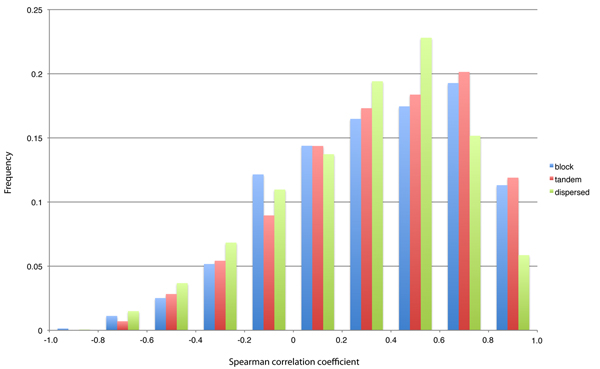
**Histogram of Spearman correlation coefficients (*ρ*) of expression**. Block pairs are indicated by blue, tandem pairs by red, and dispersed pairs by green.

To investigate whether the different types of duplication events had effects on expression pattern divergence of duplicated gene pairs, we first calculated the Spearman correlation coefficient between the two expression patterns for each pair and applied Mann-Whitney U test to assess the difference in the distributions of expression correlation (Figure 1). Both block and tandem pairs showed a significantly higher correlation in expression than dispersed pairs (both *p *values < 0.005). However, no significant difference was found in the distributions of expression correlation between block and tandem pairs (*p *value = 0.24).

**Figure 2:**
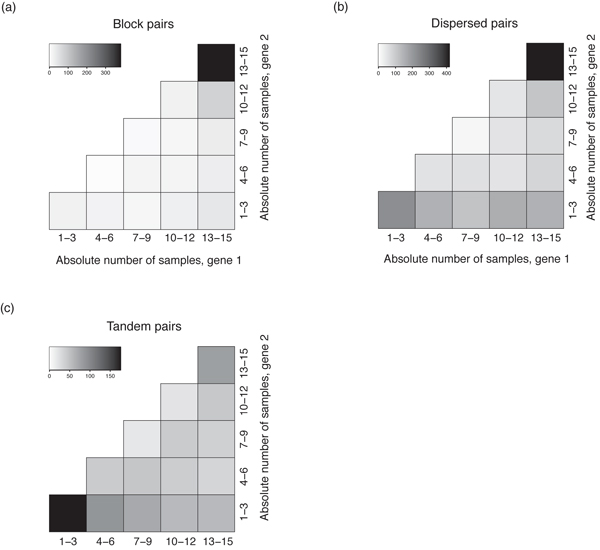
**Distribution of absolute number of samples in which duplicated gene pairs expressed**. (a) block, (b) dispersed, and (c) tandem pairs. Each cell corresponds to a range of absolute number of samples. The top-right cell represents both members of gene pairs expressed in 13–15 samples; the bottom-left cell represents both members expressed in 1–3 samples; the bottom-right cell represents gene 1 expressed in 13–15 samples and gene 2 expressed in 1–3 samples. Gray scales indicate the number of duplicated pairs within the corresponding squares. Since two members of duplicated pairs are unordered, we set the expression number of gene 1 less than that of gene 2 for each duplicated pair.

**Figure 3:**
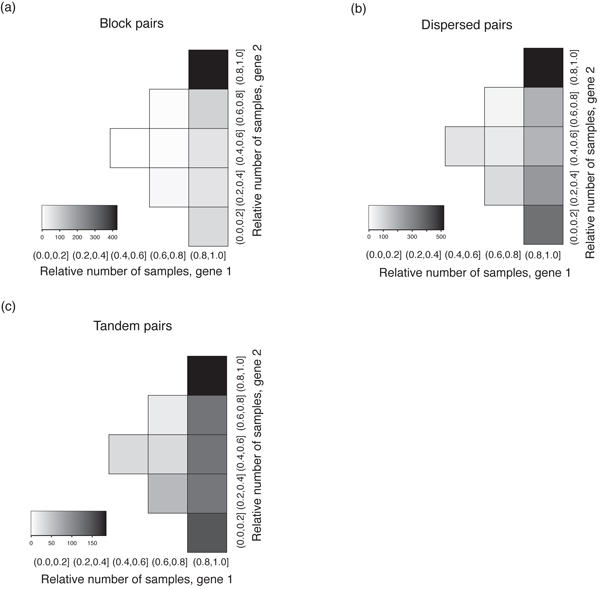
**Distribution of relative numbers of samples in which duplicated gene pairs expressed**. (a) block, (b) dispersed, and (c) tandem pairs. Each cell corresponds to a range of relative number of samples. The top-right cell represents the relative number of both members in the range of (0.8, 1.0]; the bottom-right cell represents the relative number of gene 1 in the range (0.8, 1.0] and that of gene 2 in the range of (0.0, 0.2]. Cells near the centre represent the relative number of both members close to 0.5. Gray scales indicate the number of duplicated pairs within the corresponding squares. Here we also set the expression number of gene 1 less than that of gene 2 for each duplicated pair. By the definition of relative expression number, the sum of relative expression numbers of two members should greater than or equal to one.

Next, we considered the absolute number of samples in which genes were expressed as an indicator of the expression breadth [21]. For block pairs, both members tended to express in a large number of samples (Figure 2a). Although this tendency was still recognizable for dispersed pairs, some of them had both members expressed in a small number of samples (Figure 2b). On the other hand, tandem duplicates tended to express in a small number of samples (Figure 2c). The relative expression number can be used to discriminate between redundancy, complementary and asymmetric divergence [21]. Figure 3 showed the distributions of relative number of samples for block, tandem and dispersed pairs. All three groups had a high density close to the range of (0.8, 1.0], suggesting a redundant expression mode. Nevertheless, both tandem and dispersed groups had larger proportions of pairs expressed asymmetrically and complementarily than those in the block group, and this discrepancy was more manifest for tandem duplicates (Figure 3b, c).

#### Expression divergence is correlated with synonymous substitution rate

**Figure 4:**
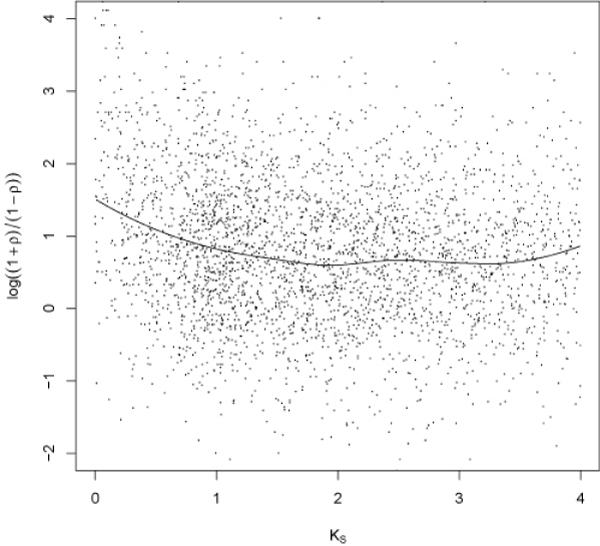
**Scatter plot of synonymous substitution rate (*K*_S_) and transformed expression correlation coefficient log [(1+*ρ*)/(1-*ρ*)]**. The solid line is the fitted curve by local regression indicating the relationship between *K*_S _and transformed correlation coefficient of expression for duplicated gene pairs.

We explored the relationship between expression divergence and synonymous substitution rate *K*_S _which is widely adopted to approximately represent divergence age [38]. Not surprisingly, a significant correlation was found between expression correlation and *K*_S _estimates for duplicated gene pairs with Spearman correlation test (*ρ *= -0.14, *p *value = 7.0 × 10^-16^). The local regression curve (Figure 4) showed that the expression correlation declined as *K*_S _increased. Moreover, such downward tendency of expression correlation was more obvious when *K*_S _was small, implying quick divergence in expression patterns after the duplication events.

### The difference in expression correlation between block and dispersed pairs could be mainly attributed to synonymous substitution rate

**Figure 5:**
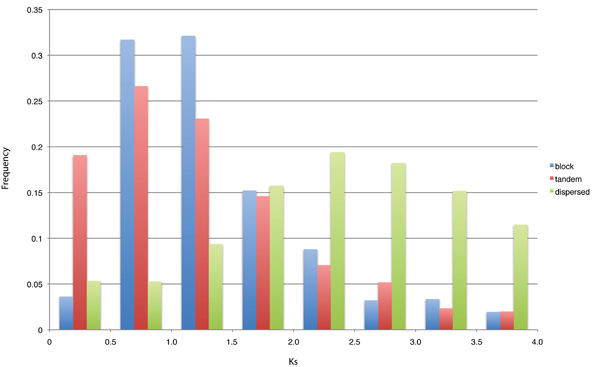
**Histogram of synonymous substitution rate (*K*_S_)**. Block pairs are indicated by blue, tandem pairs by red, and dispersed pairs by green.

Distribution of *K*_S _(Figure 5) showed that dispersed pairs had significantly higher *K*_S _values than block and tandem pairs (Mann-Whitney U test, both *p *values < 2.2 × 10^-16^), and a significant difference existed between *K*_S _of block and tandem pairs (Mann-Whitney U test, *p *value = 1.3 × 10^-6^). On the other hand, a negative correlation was observed between expression correlation and synonymous substitution rate as reported above. We further evaluated the attribution of duplication mode and *K*_S_. The conventional ANOVA was not applicable to our case since the number of gene pairs in each duplication type was extremely unbalanced. Instead, a simple linear model (formula (1)) was applied. The significance of regression coefficient can be used to indicate the attribution of the corresponding term to the difference of expression correlation. Parametric statistical test (t-test) of ordinary least square estimates (Supplementary table 1 in Additional file 1) showed that regression coefficients except for *β*_2 _and *β*_4 _were significant (*p *values < 0.001). Bootstrapping MM-estimates of coefficients (Supplementary table 2 in Additional file 1) also showed the same results, all coefficients but *β*_2 _and *β*_4 _were significant at 5% level. These results suggested that the difference in expression correlation between block and dispersed pairs could be mainly attributed to the difference of divergence time. On the other hand, the difference in expression correlation between tandem pairs and the other two types was attributed to the difference in both divergence time and duplication mode.

## Discussion

In agreement with publications on *Arabidopsis *[21], we found a significant difference between expression divergence patterns of duplicated genes derived from different duplication modes, i.e., large-scale duplication, tandem duplication and dispersed duplication. Previous studies have shown that a positive correlation between expression divergence and sequence divergence existed in duplicated genes in yeast [17], human [18] and *Arabidopsis *[21,22]. In this study, we also found a significant correlation between expression divergence and synonymous substitution rate for rice duplicated genes. In addition, we evaluated the attribution of the duplication mode and divergence time to the expression divergence of duplicated genes. Our analysis suggested that for block and dispersed pairs, divergence time played an important role in modeling the divergence of expression pattern, while duplication type had less contribution to the expression pattern divergence. On the other hand, difference in expression divergence between tandem pairs and other duplicated pairs could not be explained by divergence time only.

To obtain a reliable dataset, we applied the relative stringent criteria [31] to identify duplicated pairs and the statistical model [33] was used to detect collinear blocks in the rice genome. All pairs located in short significant collinear blocks were discarded to ensure the unambiguous classification of duplicated pair. A quality control was performed to the raw microarray data and one slide with possible artifacts was not used. Stringent criteria were adopted to filter the expression data. Due to the high divergence of rice duplicated genes [29,47], we adopted a fairly loose empirical constraint for *K*_S_(*K*_S _< 4) in this study. We also investigated the subsets of our data with various *K*_S _constraint (*K*_S _< 3.0, 3.5, 4.5, 5.0) and reached similar results. Such consistency also suggested the robustness of our results. However, some genes were present in more than one gene pairs, which violated the independent assumption of the linear model. Therefore, in addition to ordinary least square estimation and parametric statistical test, we applied robust estimation and non-parametric bootstrap to regression coefficients to obtain reliable results. Furthermore, to check the influence of such violation of assumption, we selected independent pairs following the method proposed by Gu *et. al. *[17] and performed all analyses on this subset. The analysis results of this subset were consistent with that of the whole dataset (Additional file 2).

It has long been known that gene duplication is rampant in evolution. However, the driving force behind this is still elusive. Functional complementation from duplicated genes has long been proposed as a mechanism for the emergence of gene network robustness [12]. Previous investigations have indicated the significant role of duplicated genes on genetic robustness in yeast [48] and *C. elegans *[49]. Chapman *et al. *[14] analyzed the coding sequences and SNPs between duplicated genes and singleton genes in the rice and *Arabidopsis *genomes, and proposed that buffering of critical functions may be especially important in the retention of duplicated genes. In our study, expression patterns, especially the distribution of relative numbers of expressed samples, showed that a large number of duplicated genes in all three classes expressed in a redundant or overlapping manner. This tendency was most obvious for block pairs derived by large-scale duplications, which was also reported in *Arabidopsis *[21]. Our results are consistent with previous observations and suggest that functional buffering may play an important role in the retention of duplicated genes. On the other hand, tandem genes showed a distinguished expression pattern in comparison with the other two classes of duplicated genes. The most obvious difference was the expression breadth. Many tandem pairs had both members expressed in a small number of samples. Moreover, in spite of their recent origin, tandem duplicated genes showed stronger signal for asymmetry and complementary expression than the dispersed pairs, which could not be explained by the buffering model only. Such quick divergence of recently duplicated genes could also be found in *Arabidopsis *[22]. Finally, the difference of expression pattern between tandem pairs and the other pairs suggests that neofunctionalization and subfunctionalization may play important roles in the evolution of tandem pairs.

## Conclusion

In this study, we used genome-scale expression data to examine the expression divergence patterns of rice duplicated genes. A significant difference in expression divergence patterns was revealed for duplicated gene pairs generated through different types of duplications. Furthermore, our results indicated a correlation between the expression divergence measured by the Spearman correlation coefficient and the sequence divergence measured by synonymous substitution rate. We evaluated the attribution of these two factors and found that the difference in expression divergence between block and dispersed duplicated genes can be largely explained by their different sequence divergence. On the other hand, tandem duplication showed a special expression divergence pattern in comparison with the other two types of duplicates, implying their difference in the evolutionary course. To our knowledge, this is the first attempt for a systematic investigation of expression divergence pattern for duplicated genes in the rice genome.

## Competing interests

The authors declare that they have no competing interests.

## Authors' contributions

ZL made analysis and wrote the paper. HZ made the analysis. SG and XG revised the manuscript. GG and JL supervised the project. All authors read the final manuscript.

## Supplementary Material

Additional file 1 **Estimates and statistical tests of regression coefficients**. Additional file 1 includes two tables. The first one is the ordinary least square estimates and t-tests of the regression coefficients of the linear model (1). The second table contains bootstrap confidence intervals for MM-estimates of the regression coefficients. Click here for file

Additional file 2 **Analysis of independent pairs**. This file contains all analysis results of the independent pairs. Click here for file

## References

[B1] Ohno S (1970). Evolution by Gene Duplication.

[B2] Taylor JS, Raes J (2004). Duplication and divergence: the evolution of new genes and old ideas. Annu Rev Genet.

[B3] Lynch M, Conery JS (2000). The evolutionary fate and consequences of duplicate genes. Science.

[B4] Cui L, Wall PK, Leebens-Mack JH, Lindsay BG, Soltis DE, Doyle JJ, Soltis PS, Carlson JE, Arumuganathan K, Barakat A (2006). Widespread genome duplications throughout the history of flowering plants. Genome Res.

[B5] Otto SP, Whitton J (2000). Polyploid incidence and evolution. Annu Rev Genet.

[B6] Vision TJ, Brown DG, Tanksley SD (2000). The origins of genomic duplications in Arabidopsis. Science.

[B7] Bowers JE, Chapman BA, Rong J, Paterson AH (2003). Unravelling angiosperm genome evolution by phylogenetic analysis of chromosomal duplication events. Nature.

[B8] Blanc G, Hokamp K, Wolfe KH (2003). A recent polyploidy superimposed on older large-scale duplications in the Arabidopsis genome. Genome Res.

[B9] Lynch M, Force A (2000). The probability of duplicate gene preservation by subfunctionalization. Genetics.

[B10] Stoltzfus A (1999). On the possibility of constructive neutral evolution. J Mol Evol.

[B11] Force A, Lynch M, Pickett FB, Amores A, Yan YL, Postlethwait J (1999). Preservation of duplicate genes by complementary, degenerative mutations. Genetics.

[B12] Gu X (2003). Evolution of duplicate genes versus genetic robustness against null mutations. Trends Genet.

[B13] Nadeau JH, Sankoff D (1997). Comparable rates of gene loss and functional divergence after genome duplications early in vertebrate evolution. Genetics.

[B14] Chapman BA, Bowers JE, Feltus FA, Paterson AH (2006). Buffering of crucial functions by paleologous duplicated genes may contribute cyclicality to angiosperm genome duplication. Proc Natl Acad Sci USA.

[B15] Pal C, Papp B, Lercher MJ (2006). An integrated view of protein evolution. Nat Rev Genet.

[B16] Carroll SB (2005). Evolution at Two Levels: On Genes and Form. PLoS Biol.

[B17] Gu Z, Nicolae D, Lu HH, Li WH (2002). Rapid divergence in expression between duplicate genes inferred from microarray data. Trends Genet.

[B18] Makova KD, Li WH (2003). Divergence in the spatial pattern of gene expression between human duplicate genes. Genome Res.

[B19] Blanc G, Wolfe KH (2004). Functional divergence of duplicated genes formed by polyploidy during Arabidopsis evolution. Plant Cell.

[B20] Haberer G, Hindemitt T, Meyers BC, Mayer KF (2004). Transcriptional similarities, dissimilarities, and conservation of cis-elements in duplicated genes of Arabidopsis. Plant Physiol.

[B21] Casneuf T, De Bodt S, Raes J, Maere S, Peer Y Van de (2006). Nonrandom divergence of gene expression following gene and genome duplications in the flowering plant Arabidopsis thaliana. Genome Biol.

[B22] Ganko EW, Meyers BC, Vision TJ (2007). Divergence in expression between duplicated genes in Arabidopsis. Mol Biol Evol.

[B23] Izawa T, Shimamoto K (1996). Becoming a model plant: The importance of rice to plant science. Trends in Plant Science.

[B24] Goff SA, Ricke D, Lan TH, Presting G, Wang R, Dunn M, Glazebrook J, Sessions A, Oeller P, Varma H (2002). A draft sequence of the rice genome (Oryza sativa L. ssp. japonica). Science.

[B25] Yu J, Hu S, Wang J, Wong GK, Li S, Liu B, Deng Y, Dai L, Zhou Y, Zhang X (2002). A draft sequence of the rice genome (Oryza sativa L. ssp. indica). Science.

[B26] Paterson AH, Bowers JE, Chapman BA (2004). Ancient polyploidization predating divergence of the cereals, and its consequences for comparative genomics. Proc Natl Acad Sci USA.

[B27] Paterson AH, Bowers JE, Peer Y Van de, Vandepoele K (2005). Ancient duplication of cereal genomes. New Phytol.

[B28] Wang X, Shi X, Hao B, Ge S, Luo J (2005). Duplication and DNA segmental loss in the rice genome: implications for diploidization. New Phytol.

[B29] Yu J, Wang J, Lin W, Li S, Li H, Zhou J, Ni P, Dong W, Hu S, Zeng C (2005). The Genomes of Oryza sativa: a history of duplications. PLoS Biol.

[B30] Altschul SF, Madden TL, Schaffer AA, Zhang J, Zhang Z, Miller W, Lipman DJ (1997). Gapped BLAST and PSI-BLAST: a new generation of protein database search programs. Nucleic Acids Res.

[B31] Gu Z, Cavalcanti A, Chen FC, Bouman P, Li WH (2002). Extent of gene duplication in the genomes of Drosophila, nematode, and yeast. Mol Biol Evol.

[B32] Rost B (1999). Twilight zone of protein sequence alignments. Protein Eng.

[B33] Wang X, Shi X, Li Z, Zhu Q, Kong L, Tang W, Ge S, Luo J (2006). Statistical inference of chromosomal homology based on gene colinearity and applications to Arabidopsis and rice. BMC Bioinformatics.

[B34] Thompson JD, Higgins DG, Gibson TJ (1994). CLUSTAL W: improving the sensitivity of progressive multiple sequence alignment through sequence weighting, position-specific gap penalties and weight matrix choice. Nucleic Acids Res.

[B35] Goldman N, Yang Z (1994). A codon-based model of nucleotide substitution for protein-coding DNA sequences. Mol Biol Evol.

[B36] Yang Z (1997). PAML: a program package for phylogenetic analysis by maximum likelihood. Comput Appl Biosci.

[B37] Wang HC, Hickey DA (2007). Rapid divergence of codon usage patterns within the rice genome. BMC Evol Biol.

[B38] Li WH (1997). Molecular Evolution Sunderland.

[B39] Jain M, Nijhawan A, Arora R, Agarwal P, Ray S, Sharma P, Kapoor S, Tyagi AK, Khurana JP (2007). F-box proteins in rice. Genome-wide analysis, classification, temporal and spatial gene expression during panicle and seed development, and regulation by light and abiotic stress. Plant Physiol.

[B40] Barrett T, Troup DB, Wilhite SE, Ledoux P, Rudnev D, Evangelista C, Kim IF, Soboleva A, Tomashevsky M, Edgar R (2007). NCBI GEO: mining tens of millions of expression profiles – database and tools update. Nucleic Acids Res.

[B41] Bolstad B (2005). affyPLM: Fitting Probe Level Models. Bioconductor Vignettes Release.

[B42] Gentleman RC, Carey VJ, Bates DM, Bolstad B, Dettling M, Dudoit S, Ellis B, Gautier L, Ge Y, Gentry J (2004). Bioconductor: open software development for computational biology and bioinformatics. Genome Biol.

[B43] Irizarry RA, Hobbs B, Collin F, Beazer-Barclay YD, Antonellis KJ, Scherf U, Speed TP (2003). Exploration, normalization, and summaries of high density oligonucleotide array probe level data. Biostatistics.

[B44] Yohai V (1987). High breakdown-point and high efficiency robust estimates for regression. Annals of Statistics.

[B45] Freedman D (1981). Bootstrapping regression models. Annals of Statistics.

[B46] Katju V, Lynch M (2003). The structure and early evolution of recently arisen gene duplicates in the Caenorhabditis elegans genome. Genetics.

[B47] Vandepoele K, Simillion C, Peer Y Van de (2003). Evidence that rice and other cereals are ancient aneuploids. Plant Cell.

[B48] Gu Z, Steinmetz LM, Gu X, Scharfe C, Davis RW, Li WH (2003). Role of duplicate genes in genetic robustness against null mutations. Nature.

[B49] Conant GC, Wagner A (2004). Duplicate genes and robustness to transient gene knock-downs in Caenorhabditis elegans. Proc Biol Sci.

