# Supplementary material for: Expression pattern divergence of duplicated genes in rice
Source: BMC Bioinformatics. 2009 Jun 16;10(Suppl 6):S8. doi: 10.1186/1471-2105-10-S6-S8 (PMC2697655; doi:10.1186/1471-2105-10-S6-S8)
Supplement: Additional file 1 — Estimates and statistical tests of regression coefficients. Additional file 1 includes two tables. The first one is the ordinary least square estimates and t-tests of the regression coefficients of the linear model (1). The second table contains bootstrap confidence intervals for MM-estimates of the regression coefficients. [file 1471-2105-10-S6-S8-S1.doc]

**Additional File 1**

Supplementary table 1 lists ordinary estimates and t-tests of the regression coefficients of the linear model (formula (1) in Material and methods). Supplementary table 2 contains 95% bootstrap confidence intervals for the MM-estimates of the regression coefficients.

## Supplementary Table 1 - Least square estimates of regression coefficients

This table shows the estimated coefficient, standard error, corresponding *t* statistic, and derived *p*-value for each regression coefficient in linear model (1).

|  | Estimate­­ | Standard error | t statistic | p value |
| --- | --- | --- | --- | --- |
| *β*_0_ | 0.91563 | 0.06318 | 14.491 | < 2e-16 *** |
| *β*_1_ | -0.09213 | 0.02521 | -3.654 | 0.000262 *** |
| *β*_2_ | 0.04433 | 0.10210 | 0.434 | 0.664155 |
| *β*_3_ | 0.37007 | 0.08862 | 4.176 | 3.05e-05 *** |
| *β*_4_ | 0.03991 | 0.05691 | 0.701 | 0.483138 |
| *β*_5_ | -0.18221 | 0.04849 | -3.758 | 0.000175 *** |

*** indicates *p* value < 0.001

## Supplementary Table 2 - Bootstrap confidence intervals for regression coefficients

95% bootstrap confidence intervals were derived for MM-estimates of regression coefficients. Four standard methods were used: the basic bootstrap interval, the studentized bootstrap interval, the bootstrap percentile interval, and the adjusted bootstrap percentile (BCa) interval.

|  | Basic | Normal | Percentile | BCa |
| --- | --- | --- | --- | --- |
| *β*_0_ | (0.7673, 1.0245) * | (0.7650, 1.0245) * | (0.7637, 1.0208) * | (0.7651, 1.0235) * |
| *β*_1_ | (-0.1364, -0.0360) * | (-0.1365, -0.0345) * | (-0.1344, -0.0340) * | (-0.1355, -0.0348) * |
| *β*_2_ | (-0.5262, 0.0583) | (-0.5283, 0.0544) | (-0.5344, 0.0500) | (-0.5346, 0.0494) |
| *β*_3_ | (0.1643, 0.5413) * | (0.1320, 0.7575) * | (0.1620, 0.5390) * | (0.1612, 0.5378) * |
| *β*_4_ | (-0.0448, 0.1974) | (-0.0451, 0.1962) | (-0.0476, 0.1946) | (-0.0468, 0.1961) |
| *β*_5_ | (-0.2878, -0.0829) * | (-0.2891, -0.0810) * | (-0.2864, -0.0815) * | (-0.2873, -0.0821) * |

* indicates the significance of the corresponding regression coefficient in the bootstrap procedure
