# Supplementary material for: Expression pattern divergence of duplicated genes in rice
Source: BMC Bioinformatics. 2009 Jun 16;10(Suppl 6):S8. doi: 10.1186/1471-2105-10-S6-S8 (PMC2697655; doi:10.1186/1471-2105-10-S6-S8)
Supplement: Additional file 2 — Analysis of independent pairs. This file contains all analysis results of the independent pairs. [file 1471-2105-10-S6-S8-S2.doc]

**Additional File 2**

Independent pairs were selected following the approach proposed by Gu *et al.* (ref [17]). A total number of 1,287 independent pairs were obtained. This subset contained 618 block pairs, 281 tandem pairs and 388 dispersed pairs. Mann-Whitney U test showed that expression correlation and synonymous substitution rate *K*_S_ of three types of duplicated pair were significant different (*p* value < 0.01; Supplementary figures 1 and 2). *K*_S_ and expression correlation were significantly correlated (Spearman correlation test, *ρ* = -0.19, *p* value = 1.72×10^-11^; Supplementary Figure 3). Ordinary least square estimates and t-tests of coefficients of the linear model (formula (1) in Materials and Methods) are listed in Supplementary table 3. Supplementary table 4 shows the bootstrap confidence intervals for each regression coefficient. The results were consistent with those of full dataset. For the selection of independent pairs, preference for closely related duplicated genes causes information loss of highly diverged duplicated genes and results in altered sample distribution. *K*_S_ values of independent pairs were significantly lower than those of full dataset (Mann-Whitney U test *p* value < 2.2×10^-16^, two-tailed). The influence of altered sample distribution showed in the significant difference in expression correlation between block and tandem duplicates, and the significance of *β*_4_. The consistent patterns were observed for absolute and relative expression numbers, even with finer differences in the exact numbers for each cell (Supplementary figures 4 and 5).

# Figures

## Supplementary Figure 1 - Histogram of Spearman correlation coefficients of expression for independent pairs.

Block pairs are indicated by blue, tandem pairs by red, and dispersed pairs by green.


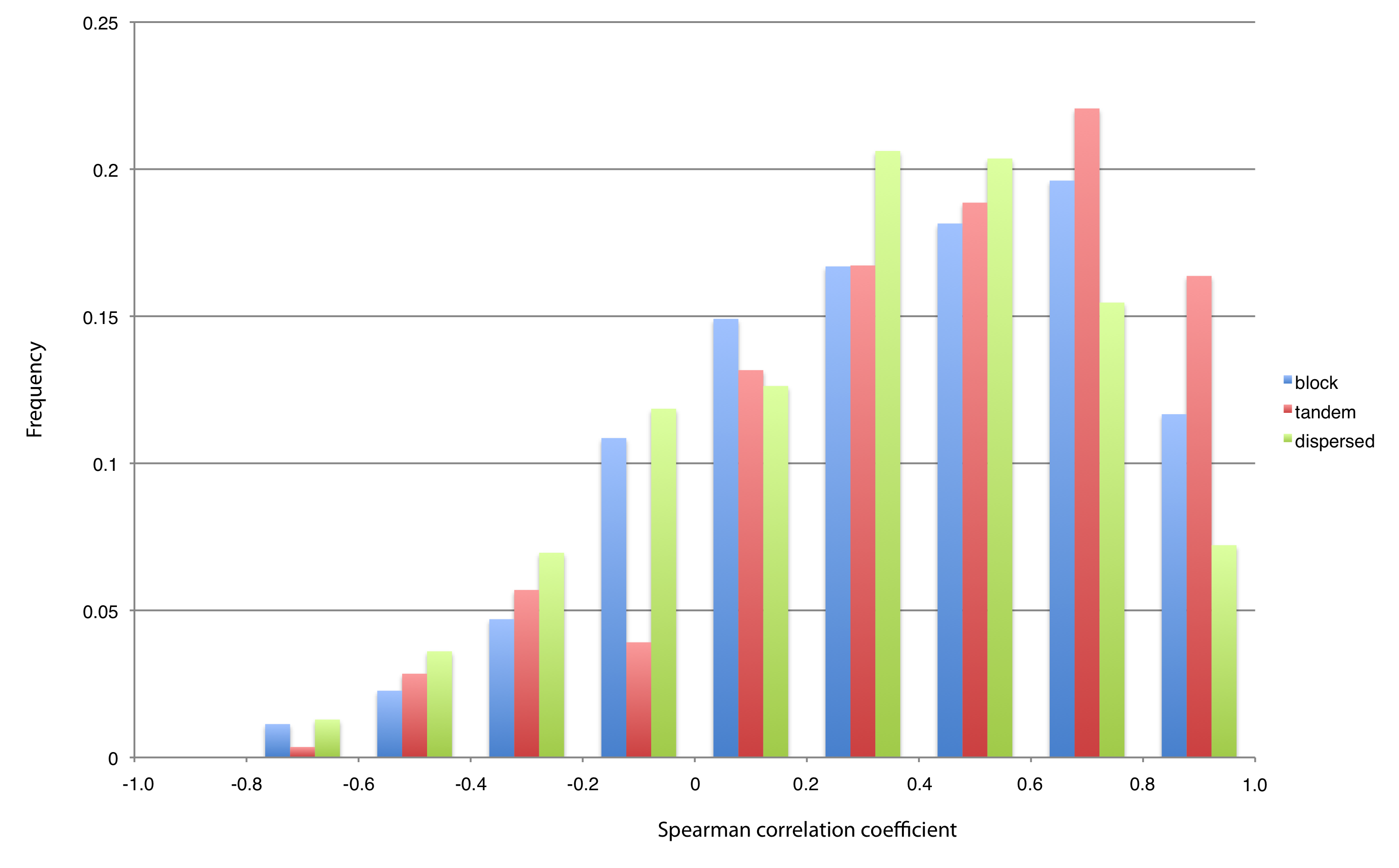


## Supplementary Figure 2 - Histogram of synonymous substitution rate (*K*_S_) for independent pairs.

Block pairs are indicated by blue, tandem pairs by red, and dispersed pairs by green.


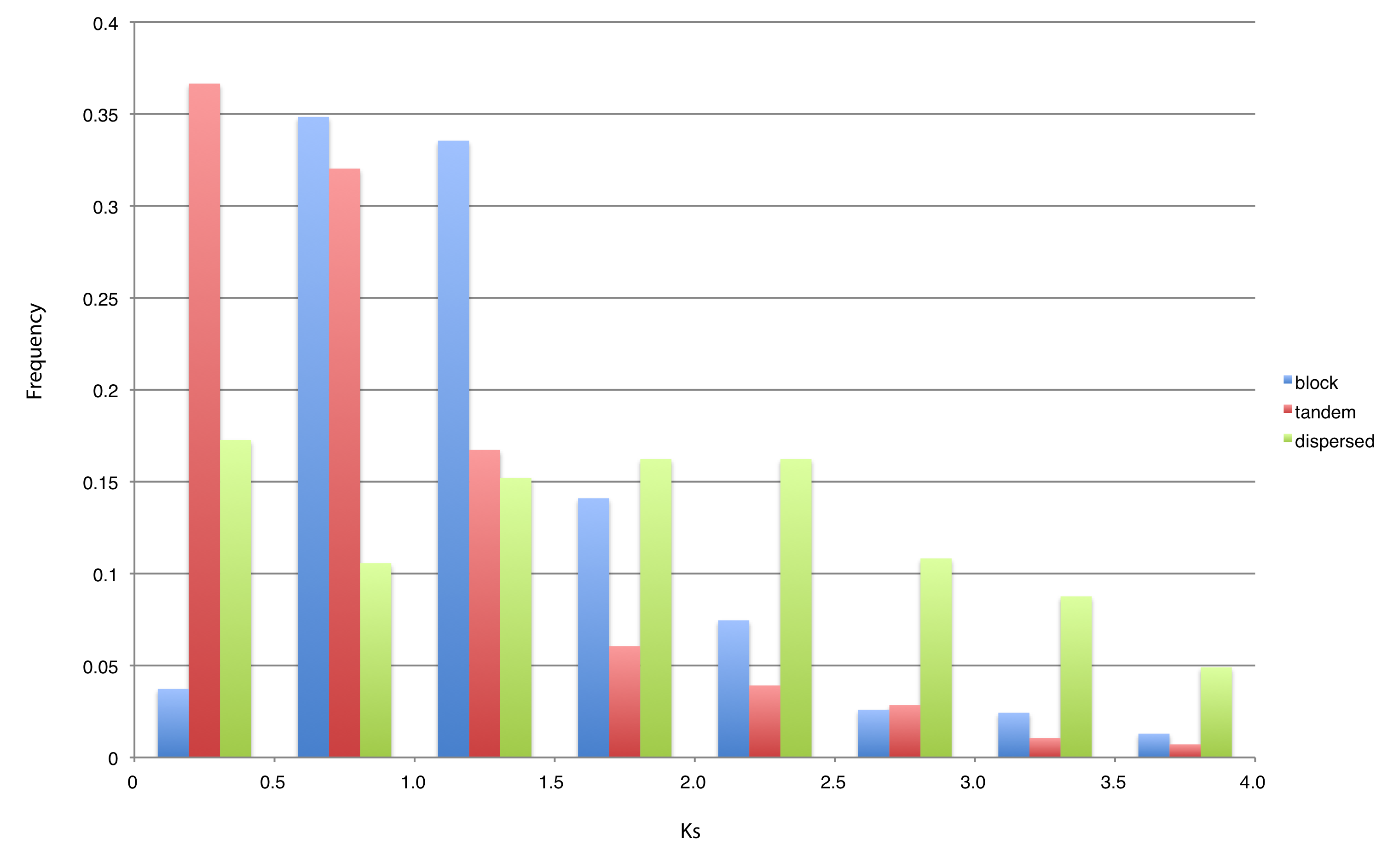


## Supplementary Figure 3 - Scatter plot of synonymous substitution rate (*K*_S_) and transformed expression correlation coefficient log[(1+*ρ*)/(1-*ρ*)].

The solid line is the fitted curve by local regression indicating the relationship between *K*_S_ and transformed correlation coefficient of expression for independent pairs.


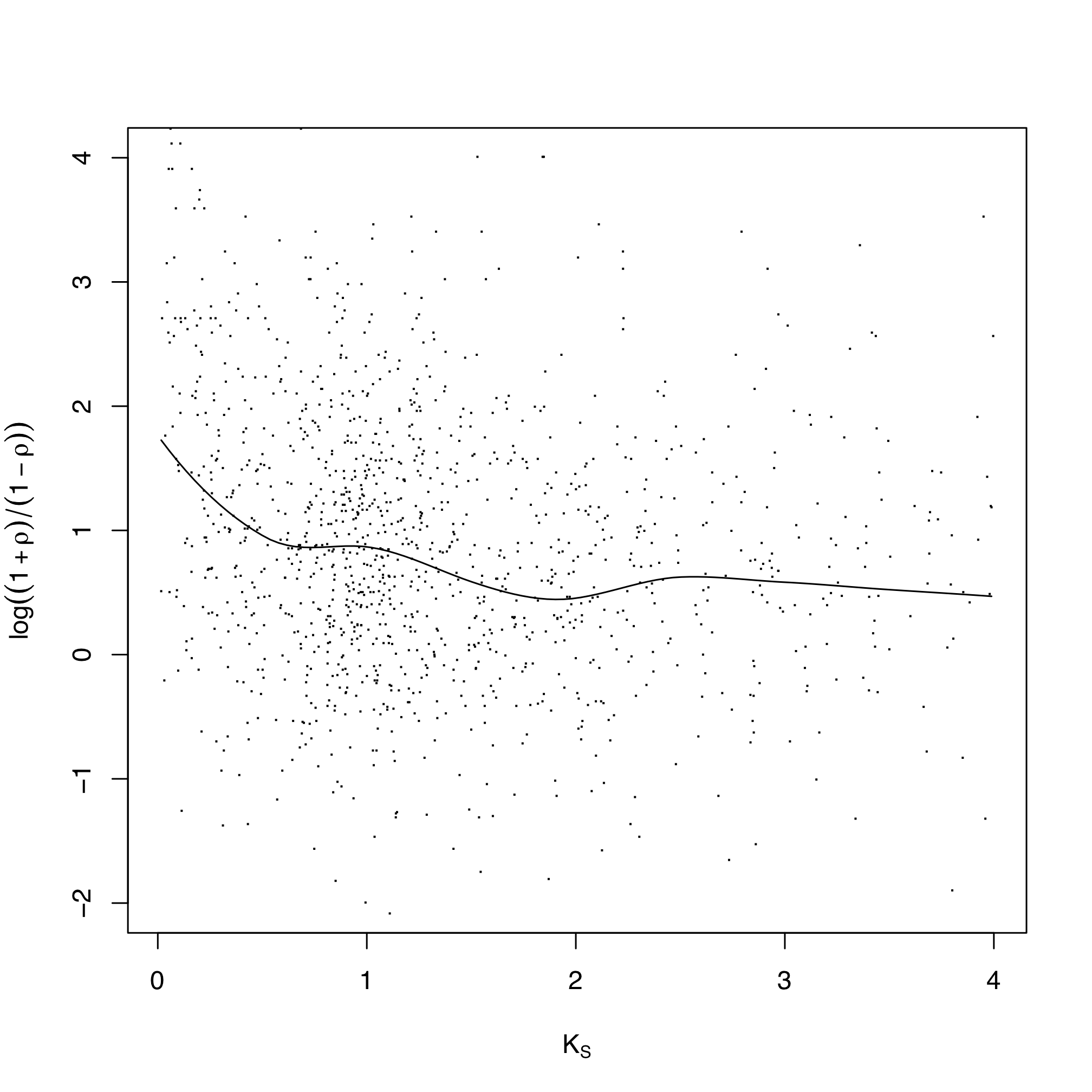


## Supplementary Figure 4 - Distribution of absolute number of samples in which independent duplicated gene pairs expressed.

(a) block, (b) dispersed and (c) tandem pairs. Each cell corresponds to a range of absolute number of samples. The top-right cell represents both members of gene pairs expressed in 13-15 samples; the bottom-left cell represents both members expressed in 1-3 samples; the bottom-right cell represents gene 1 expressed in 13-15 samples and gene 2 expressed in 1-3 samples. Gray scales indicate the number of duplicated pairs within the corresponding cells. Since two members of duplicated pairs are unordered, we set the expression number of gene 1 less than that of gene 2 for each duplicated pair.


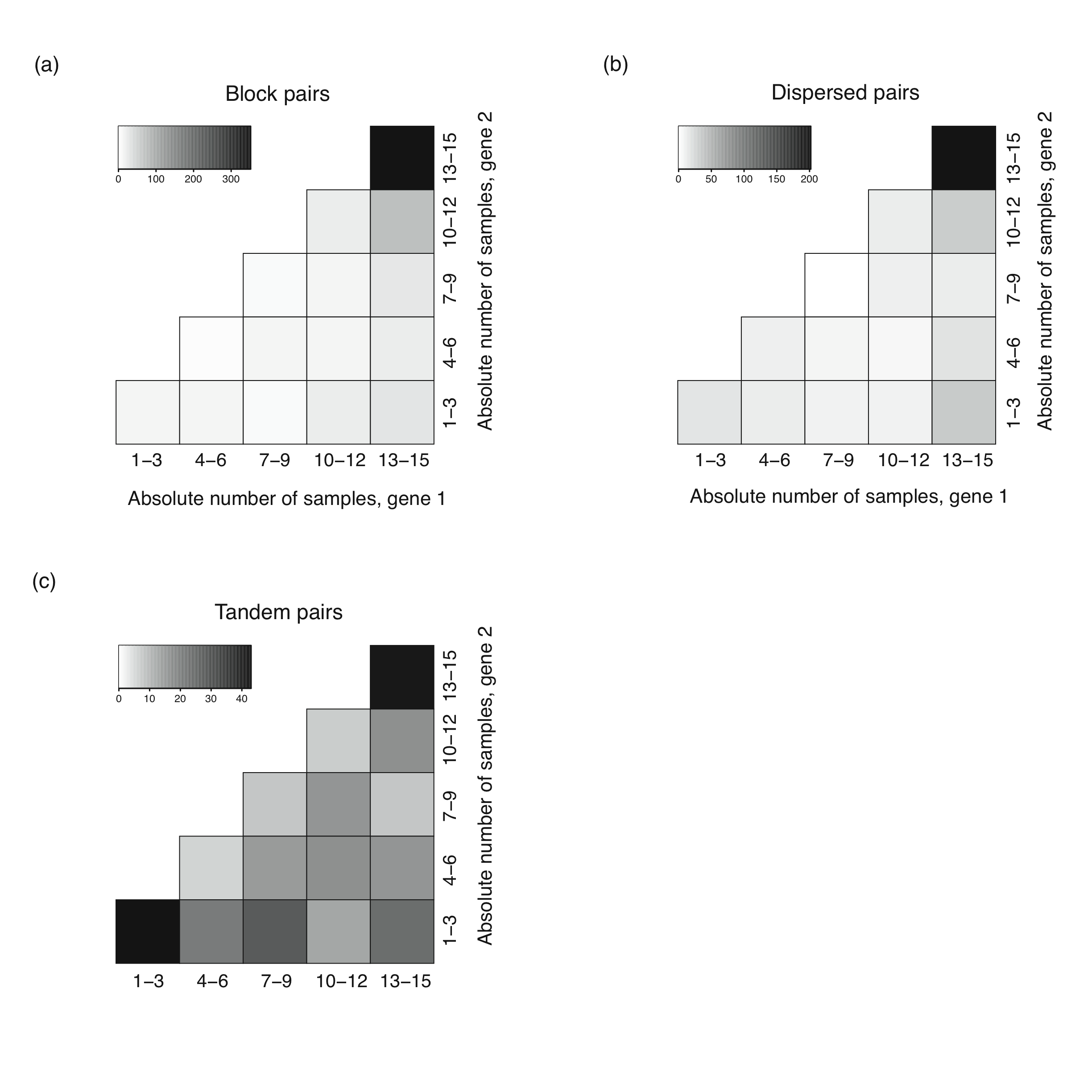


## Supplementary Figure 5 - Distribution of relative numbers of samples in which independent duplicated gene pairs expressed.

(a) block, (b) dispersed, and (c) tandem pairs. Each cell corresponds to a range of relative number of samples. The top-right cell represents the relative number of both members in the range of (0.8, 1.0]; the bottom-right cell represents the relative number of gene 1 in the range (0.8, 1.0] and that of gene 2 in the range of (0.0, 0.2]. Cells near the centre represent the relative number of both members close to 0.5. Gray scales indicate the number of duplicated pairs within the corresponding cells. Here we also set the expression number of gene 1 less than that of gene 2 for each duplicated pair. By the definition of relative expression number, the sum of relative expression numbers of two members should greater than or equal to one.


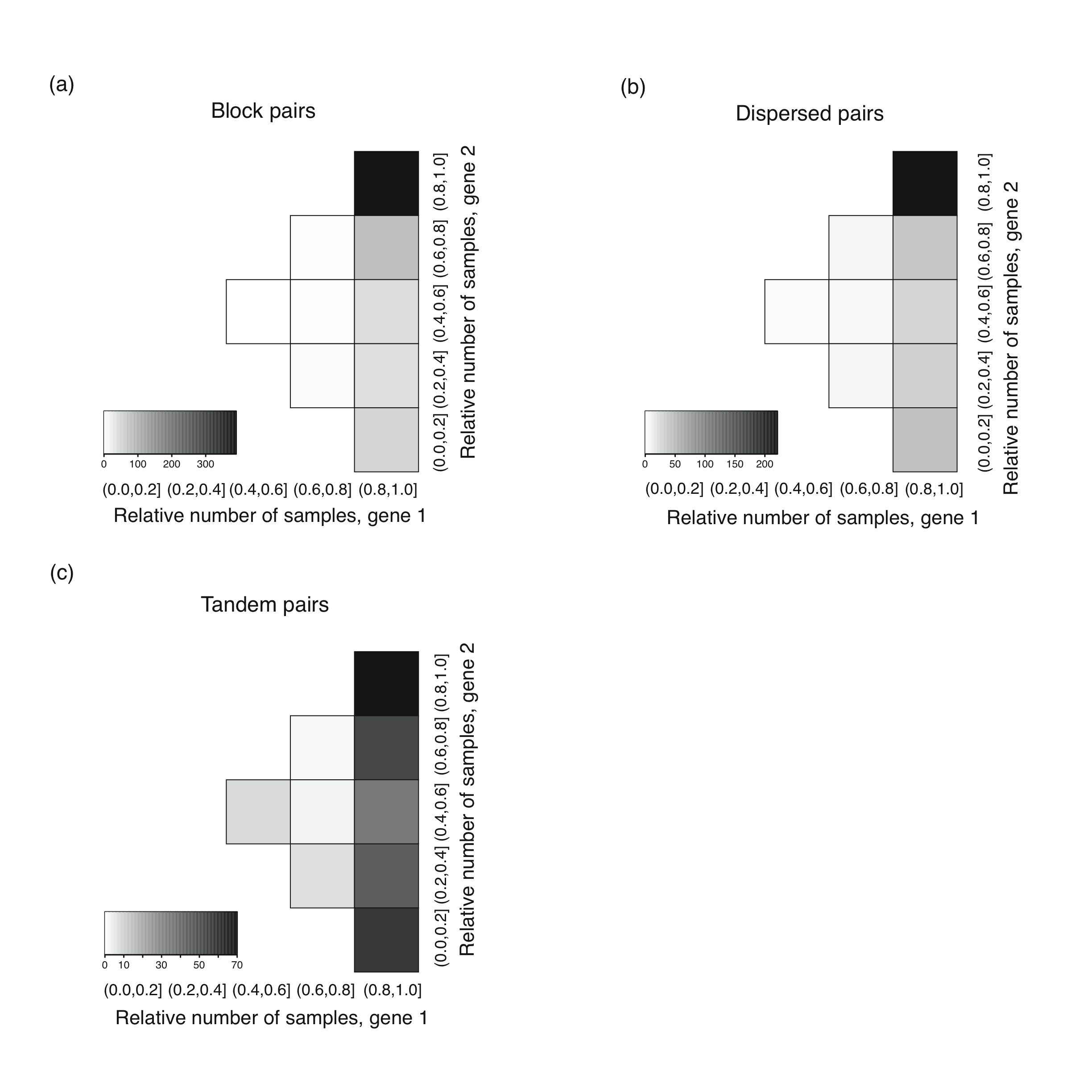


# Tables

## Supplementary Table 3 - Bootstrap confidence intervals for regression coefficients for independent pairs

This table shows the estimated coefficient, standard error, corresponding *t* statistic, and derived *p*-value for each regression coefficient in formula (1).

|  | **Estimate** | **Standard error** | **t value** | **p value** |
| --- | --- | --- | --- | --- |
| *β*_0_ | 1.08471 | 0.10401 | 10.428 | < 2e-16*** |
| *β*_1_ | -0.21005 | 0.05124 | -4.100 | 4.4e-05*** |
| *β*_2_ | -0.14675 | 0.13943 | -1.053 | 0.29277 |
| *β*_3_ | 0.42684 | 0.14413 | 2.961 | 0.00312** |
| *β*_4_ | 0.20531 | 0.08121 | 2.528 | 0.01158* |
| *β*_5_ | -0.2201 | 0.10261 | -2.145 | 0.03214* |

* indicates *p* value < 0.05; ** indicates *p* value < 0.01; *** indicates *p* value < 0.001

## Supplementary Table 4 - Bootstrap confidence intervals for regression coefficients for independent pairs

95% bootstrap confidence intervals were derived for MM-estimates of regression coefficients. Four standard methods were used: the basic bootstrap interval, the studentized bootstrap interval, the bootstrap percentile interval, and the adjusted bootstrap percentile (BCa) interval.

|  | **Normal** | **Basic** | **Percentile** | **BCa** |
| --- | --- | --- | --- | --- |
| *β*_0_ | (0.844, 1.264)* | (0.842, 1.265)* | (0.841, 1.265)* | (0.844, 1.268)* |
| *β*_1_ | (-0.2996, -0.1020)* | (-0.2977, -0.1001)* | (-0.3022, -0.1046)* | (-0.3014, -0.1037)* |
| *β*_2_ | (-0.5283, 0.0544) | (-0.5262, 0.0583) | (-0.5344, 0.0500) | (-0.5346, 0.0494) |
| *β*_3_ | (0.1320, 0.7575)* | (0.1260, 0.7601)* | (0.1288, 0.7629)* | (0.1306, 0.7664)* |
| *β*_4_ | (0.0844, 0.4109)* | (0.0827, 0.4082)* | (0.0885, 0.4139)* | (0.0903, 0.4181)* |
| *β*_5_ | (-0.4613, -0.0210)* | (-0.4603, -0.0172)* | (-0.4640, -0.0204)* | (-0.4620, -0.0189)* |

* indicates the significance of the corresponding regression coefficient in the bootstrap procedure
